# The power of feeling seen: perspectives of individuals with eating disorders on receiving validation

**DOI:** 10.1186/s40337-021-00500-x

**Published:** 2021-11-08

**Authors:** Josie Geller, A. Fernandes, S. Srikameswaran, R. Pullmer, S. Marshall

**Affiliations:** 1 St. Paul’s Hospital Eating Disorders Program, 1081 Burrard Street, Vancouver, BC V6Z 1Y6 Canada; 2 Department of Psychiatry, University of British Columbia, Vancouver, Canada; 3 Department of Adolescent Health and Medicine, University of British Columbia, Vancouver, Canada

**Keywords:** Validation, Eating disorders, Self-compassion, Recovery

## Abstract

**Background:**

A common complaint of individuals suffering from mental health conditions is feeling invalidated or misunderstood by care providers. This is notable, given that non-collaborative care has been linked to poor engagement, low motivation and treatment non-adherence. This study examined how receiving validation from care providers is experienced by individuals who have an eating disorder (ED) and the impact of receiving validation on the recovery journey.

**Methods:**

Eighteen individuals who had an eating disorder for an average duration of 19.1 years (two identifying as male, 16 identifying as female), participated in semi-structured interviews on barriers and facilitators to self-compassion. Seven were fully recovered, and 11 were currently participating in recovery-focused residential treatment. Thematic analysis focused on the meaning and impact of receiving validation to participants.

**Results:**

Five care provider actions were identified: (i) making time and space for me, (ii) offering a compassionate perspective, (iii) understanding and recognizing my treatment needs, (iv) showing me I can do this, and (v) walking the runway. These were associated with four key experiences (feeling trust, cared for, empowered, and inspired), that participants described as supportive of their recovery.

**Conclusions:**

This research provides insight into patient perspectives of validation and strategies care providers can use, such as compassionate reframing of difficult life experiences, matching interventions to patient readiness, and modeling vulnerability.

## Plain English Summary

Feeling validated (or feeling understood and accepted) is an important aspect of a patient’s experience with health care providers. The purpose of this research was to learn about the role of validation in eating disorders treatment from patients’ perspectives, and to learn how the experience of validation supports recovery from an eating disorder. In this research, interviews were conducted with eighteen individuals who were either currently seeking intensive treatment for an eating disorder or had recently recovered. Five care provider actions were identified as engendering feelings of validation: (i) making time and space for me, (ii) offering a compassionate perspective, (iii) understanding and recognizing my treatment needs, (iv) showing me I can do this, and (v) walking the runway. These actions were associated with four key patient experiences: feeling trust, cared for, empowered, and inspired. Recommendations for care providers to practice validation are made based on study findings.

## Introduction

Validation has been defined as “being awake to, accurately reflecting, and conveying acceptance of a patient’s behavior, thoughts, or feelings” [1]. It has been conceptualized as the root of empathy and compassion [2] and is described as a key contributor to the therapeutic alliance across treatment modalities, including Compassion Focused Therapy (CFT; [3]), Cognitive Behaviour Therapy (CBT; [4]) and Dialectical Based Therapy (DBT; [5]). Validation is a fundamental component of embodied therapeutic presence, or the ability to tap into emotions and experiences, foster a sense of spaciousness or larger perspective of what is happening, and being with and for others in the service of healing [6]. Finally, receiving validation over time has been linked to secure attachment, and to the development of self-compassion [5, 7], the latter of which has been shown to play a positive role in recovery from several physical and mental health conditions, including eating disorders (EDs).

There are three mechanisms by which validation has been proposed to promote recovery in the treatment of mental health conditions. It may increase the stability of self-views by helping to define and organize experiences and improve one’s ability to navigate difficult social interactions [8, 9]. This may be particularly helpful for individuals with EDs, who have been characterized as having higher levels of attachment insecurity [10]. Validation has also been described as reducing emotional arousal and enhancing learning by decreasing feelings of overwhelm associated with critical self-evaluations [11]. Finally, validation has been posited to increase motivation to stay in treatment by providing an empathic and empowering means of reflecting upon one’s personal challenges or circumstances, which patients experience as rewarding (e.g., [1]). Given the importance of motivation in recovery from an ED, validation thus has a particularly important role to play in this population. Within a DBT framework, validation is described as particularly important in treating conditions such as EDs, where symptoms may have developed to regulate affect in response to lifetime experiences with an invalidating environment [12]. That is, validation is described as helping make sense of past circumstances that were experienced as confusing and/or shaming, providing a new, self-compassionate perspective that allows for the cultivation and practice of more helpful ways of interacting with the world.

Although validation has long been recognized as a central therapeutic skill in ED treatment, not all care providers are trained in its delivery or consistently use it in practice. This may be due to validation being more of a process than content skill, reflecting the “how,” as opposed to the “what” of interventions. Several studies have shown, for instance, that it is common for both adolescent and adult patients with EDs to experience their care providers as directive and unresponsive to their needs and desires [13, 14]. Possibly, this is due to a focus of interventions in this population on outcome (e.g., behaviour change) rather than on relationship, connection, and understanding the function of behaviours. In the absence of validation, patients may have difficulty trusting that care providers will respond in a helpful way if they disclose personal difficulties. This in turn may lead to less willingness to be open, reducing their ability to understand their experiences and benefit from therapy. Indeed, in contrast to directive, unresponsive care, collaborative, responsive interventions have been linked to higher rates of treatment acceptability and retention, lower rates of dropout, and better symptom improvement [15–17]. Thus, increasing care providers’ ability to offer validation is likely to have a number of therapeutic benefits.

It is noteworthy that the literature on validation has focused primarily on its definition and delivery by clinicians, with little attention paid to patient experiences of receiving validation. Given that previous research has shown that patient ratings (rather than care provider ratings) of care provider behaviour, were predictive of outcome [18], determining what is meant by and important about validation from the perspective of patients is particularly important.

The purpose of this research was to explore how current and former patients with EDs define and experience validation, and to learn the impact of validating interventions on their recovery. Our work was guided by the following question: How do current and former patients with EDs describe experiences of receiving validation during their recovery? In order to do this, data collected from a larger study on self-compassion and the ED recovery journey were used. It is hoped that with this knowledge, specific recommendations can be offered to guide care providers on how to increase validating interactions with individuals who have EDs.

## Methods

### Participants

Participants were excluded from the study if they were under 19 years of age, receiving acute medical or psychiatric treatment, or unable to attend a 90-min interview in person. Upon confirming study eligibility, participants completed informed consent and an online survey to obtain demographic information and assess eating pathology using the Eating Disorders Examination Questionnaire (EDE-Q 6.0; [19]). Participants then completed a semi-structured audio-recorded interview (conducted by JG) aimed at exploring conceptualizations of self-compassion, barriers and facilitators to developing self-compassion, and key aspects of individual’s ED recovery journeys. A visual timeline was constructed by the participant and interviewer. The interviewer provided a template consisting of an anchor point denoting the day of the interview and the participant was prompted to recall experiences leading to the present day, and to envisage future desired experiences. This timeline was used to provide context and to record temporal relations among described experiences. The average interview length was 70 min. Following completion of the interview, participants were compensated with a $40 honorarium.

The final sample consisted of 11 current and 7 recovered patients (*N* = 18). The majority of the sample identified as female (*n* = 16) and two participants identified as male. Seventeen identified as Caucasian, and one as biracial. Participants ranged in age from 21 to 65 years (*M* = 36.65), with an average duration of illness of 19.71 years (*SD* = 13.08). At initial assessment, eight participants (44%) received a diagnosis of anorexia nervosa, five (28%) a diagnosis of bulimia nervosa, two (11%) a diagnosis of binge eating disorder and three (17%) a diagnosis of other specified feeding or eating disorder. Most of the sample experienced low levels of ED symptoms at the time of participation, with only two participants (both current patients) scoring above the EDE-Q clinical cut-off [20]. Global EDE-Q scores between the two groups (recovered and current), indicated that current patients (*M* = 3.20, *SD* = 0.72) had significantly higher levels of eating pathology than recovered individuals (*M* = 1.73, *SD* = 0.60), *t*(16) = 4.10, *p* < 0.001.

### Authors/researchers

Three authors are registered clinical psychologists working in both tertiary level adult eating disorder care and academic settings, one of whom conducted all of the interviews. One author is a research assistant with an undergraduate degree in psychology and experience working in tertiary level ED treatment programs. One author is a professor in an academic setting who regularly collaborates with health care researchers in EDs and adolescent health.

### Procedure

All procedures were approved by the Research Ethics Board where the study took place. Both current and recovered patients were purposefully sampled in order to include individuals at different points in recovery, or levels of readiness. Current patients were recruited directly from a specialized hospital-based or residential treatment program for adults with severe EDs. Three clinicians from the program identified recovered patients (i.e., those who had graduated from residential day treatment, maintained a minimum body mass index of 19.5, and did not engage in bingeing or compensatory behaviours more than once per month). Both current and recovered patients were provided with study information sheets and contacted the research coordinator to determine eligibility if interested in participating.

Data were obtained from a larger study that used a semi-structured interview focused on self-compassion in the recovery journey [21]. For the purposes of this research, all completed interviews were re-analyzed to focus on the role of validation in the ED recovery process.

### Data analyses

Latent thematic analysis [22] was employed and involved a recursive method of review. The coding team was comprised of the first four authors (JG, AF, RP, and SS) who consulted with the final author (SM) throughout the data analysis process.

**Fig. 1:**
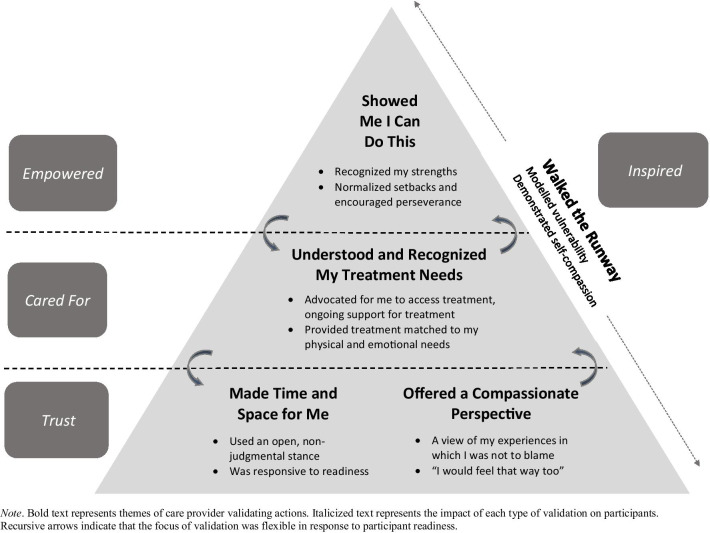
Validation themes and key experiences

The coding team read all interview transcripts and recorded initial observations. Following the first round of analysis, two separate investigations were identified: (1) patient perceptions of the power of validation, which was the primary focus of the current study, and (2) patient recovery journeys [21]. After the first exploratory round of analysis and coding, the team re-read each transcript with the following objectives: (1) What did people say or do that helped participants overcome barriers to self-compassion in the course of their recovery journeys? and (2) *Why* did validation matter to participants? What about validating experiences was important and what was the impact on their recovery journeys? A list of examples and codes were identified and codes were continually refined through data comparison within and across cases. In two subsequent rounds of interpretive analysis, team members identified patient perceptions of care provider actions that were described as validating, coded the actions as categories, and grouped these into latent themes. Participants’ key experiences resulting from their perceptions of validation were also identified and named through discussion. These discussions involved frequent reference to the participants’ timelines and narratives. Following each of three rounds of interpretive analysis, the team met and overlapping themes and key experiences were identified and condensed. After the fourth round of analysis, consensus was reached on the latent themes, which depict care provider actions. Consensus was also reached on key participant experiences associated with each theme, which depict the impact of these actions on participants. Two team members (JG and AF) created a figure (Fig. 1) based on codes and discussions to depict the themes, key experiences, and their relations with one another. The larger group discussed the figure and it was revised accordingly.

## Results

Perceived care provider actions experienced as validating were captured in five latent themes, which occurred at various points along the recovery journey, representing different levels of participant readiness. The themes were associated with four key participant experiences, proposed as central to overcoming barriers to self-compassion. As shown in Fig. 1, the first two themes, *made time and space for me*, and *offered a compassionate perspective* were associated with the key experience of feeling *trust.* The second theme, *understood and recognized my treatment needs* was associated with feeling *cared for.* The theme *showed me I can do this* was associated with feeling *empowered,* and the fifth theme, *walked the runway,* was associated with being *inspired.* Each participant key experience is described below, along with the associated perceived care provider action theme(s).

### Trust

The key experience of feeling *trust* was evoked by care provider actions that showed commitment to listening to participants’ stories and responding in a manner that made them feel understood, seen and safe. There were two themes:

*Made time and space for me*. This theme was characterized by interactions in which participants described care providers as curious about their experiences and willing to pay attention and be present. Central to this theme was attentive listening, taking time to understand, and not intervening too quickly:

“We all talk about how when people talk to us we think it’s our job to fix it, but really that’s not what people want you to do, they just want you to be like ‘that sucks’ or ‘I feel you’” Participant 4 (P4).

Participants emphasized the importance of their narrative being heard without judgment, and thus being able to discuss their experiences without negative consequences: “The feeling is relief, um I guess kind of like you feel you can be yourself almost… almost no fear of judgment from others” (P17) and a first glimmer of acceptance, or not being flawed or wrong, “Of course, there’s just like the validation of like, of course you would feel that way” (P5). Overall, the theme *making time and space for me* reflected participants’ feeling that they could speak freely and openly without having to carefully self-monitor or be at risk of being judged.

*Offered a compassionate perspective*. A second theme that contributed to feeling *trust* involved care providers offering an alternative to previously held, self-critical ways of thinking. Hearing a view of their narratives in which they were not to blame for their difficulties appeared to open participants to the possibility of a different way of relating to themselves and to a different type of relationship with the care provider:

“When it first started I think it maybe took root in (my therapist’s) office um… she helped me in those days and didn’t put the finger of guilt on me that it (my ED) was my fault and get my willpower together and I think it started there… I was able to trust, not that she’d look after me but, I began to trust that she had some understanding. She got something about me; I never felt before that anyone got me.” (P8)

Another participant explicitly described how validation allowed her to reframe responsibility for past negative experiences:

“In my own head like I mostly think that a lot of stuff that happened to me was my fault? So to um, or that I could have done something different for it not to be, for it to have been prevented. So um… validating… that wasn’t right, you didn’t choose for that to happen, uh validating this is sad, you’re disappointed, you’re you know, validating my feelings. Really it just allowed that space.” (P10)

One participant described how compassion and acceptance provided a safe space where she felt increasingly comfortable to discuss difficult past experiences:

“And so when I relay a story… I can just sense there is something that’s flowing from (my therapist), emanating from her. I guess it’s compassion. And acceptance. That makes it so safe to tell her more, or to consider the possibility. Cause it’s taken me so long to develop trust and safety. And every time I give her a little bit more, another piece, that feels difficult to do or risky to do, she has a way of holding it so tenderly I guess. That suddenly it’s not as shameful anymore.” (P12)

The benefits of being *offered a compassionate perspective* were effectively summarized by one participant who said “if they can have compassion for me, maybe I can too.” (P6).

### Cared for

A second key experience was characterized by participant descriptions of care provider actions that matched their needs, contributing to the experience of feeling *cared for*. Interestingly, in some cases this involved advocating for access to a higher level of care while in others it involved providing support to step away from treatment that was not suited to their needs at the time.

*Understood and recognized my needs*. Participants highlighted the benefits of health care providers assisting them in accessing treatment:

“After these key milestones where I told people (about my ED)… just their reactions really helped enable a sense of increased self-compassion… even like my GP, like, as awkward as that was he took it very seriously… Like there was, there was actually a really key moment when I think I needed… it was a letter I need for work about going into (the inpatient unit)… once I saw the letter I was like, there was a lot of validation with that and all of a sudden this became a real thing… this is the first thing that kinda said like, the medical community, you know, says that I have a legitimate sickness…” (P6)

Some participants described how acknowledgement from health care providers regarding the severity of their illness helped counteract feelings of being unworthy or undeserving of help:

“One of the team leaders, group leaders, she had said um, have you ever considered going into hospital? And it was the first time someone had acknowledged that I was deserving of care. And I, I thought yes I have thought, I’ve been waiting for permission. Um, for someone to, for someone to acknowledge me. And then I was able to acknowledge myself.” (P7)

One participant highlighted how her family doctor’s reaction towards her ED instilled a sense of hopefulness that she could recover if she were to seek appropriate care:

“So (my family doctor) was very thorough, he was very compassionate, and non-judgmental, like he was very validating. And then when he explained to me why he felt I needed to be on a medical ward, even though I didn’t agree with him, it felt like oh, he thinks this is a real thing. So it, it felt like maybe there was a bit of hope, and that if it was genuinely an ED then I could be helped.” (P12)

Another participant noted the impact of being offered choices to step out of treatment without risk of consequences from care providers:

“… there wasn’t the expectation that um, or I wasn’t made to feel like I was letting anybody down if I were to choose not to go further. Um, and I think that was really important because that actually helped me to decide to go further…. if I felt more pressure I would have been like, no I’m just gonna walk away cause this is too much, the stakes are too high, the chance of failing is too great …” (P5)

Thus, throughout the recovery journey, when care providers recognized participants’ health care needs, readiness, and were responsive in offering choices without risk of negative consequences, participants described feeling *cared for*.

### Empowered

Participants described feeling *empowered* to actively work on self-compassion and recovery by validating care provider comments that recognized their abilities and that conveyed confidence in their capacity to overcome setbacks:

*Showed me I can do this.* For some participants, feeling *empowered* occurred in response to care providers recognizing their strengths:

“They encourage you to take chances, and I don’t mean it in the sense of ‘here’s the goal I need you to do this week’, it’s more in the sense of helping you see things within you so that you can do stuff. It’s like empowerment, I guess. Helping you see just how capable you are… I think it’s like teaching me how to fish instead of giving me the fish or whatever?... So helping me believe in myself that I can do it.” (P17)

For others, care providers’ ongoing expressions of faith and encouragement were described as most important:

“He was very compassionate. Like I hear from some, their (family doctors) are, they say unhelpful things, he said, he kept believing in me, he kept, with great care he just kept encouraging me and kept having confidence that things could improve.” (P12)

Finally, participants noted the importance of empowering care provider behaviours at all stages of the recovery journey, including times when they experienced lapses:

“Saturday they came and visited the kids and it was great by all accounts but it was also very stressful and I ended up engaging in symptoms that evening, you know? But when I was deconstructing this in post-group on Sunday, you know, um, someone pointed out like, that like, you know, the way I was talking about it was like, ya I did but that’s ok, because you know, it’s not perfect, but I still, I had a lot of victories that day and whatever, right? And that’s apparently very different than how I used to talk about this kind of stuff. So, so I wasn’t focusing on the failures that much you know?” (P6)

In sum, participants felt *empowered* when they perceived care providers to be highlighting their strengths, conveying faith in their capacity for change, and reframing difficulties as a normal part of recovery.

### Inspired

Finally, throughout the recovery journey, participants described the positive impact of care providers sharing personal experiences in which they were vulnerable. Learning about their humanity, as well as their willingness to practice self-compassion at these times was associated with participants feeling *inspired*:

*Walking the runway.* Participants described how care provider disclosure of vulnerability helped to normalize their own difficulties. One participant highlighted the benefit of discovering that she was not alone in making mistakes or in feeling difficult emotions, such as embarrassment:

“I also really connected when care providers or people would disclose a little about themselves, their own, or even just make a comment, like an off the cuff comment about something, a mistake they’d made or something. Just seeing people talk about their mistakes as if it was nothing or no big deal was helpful because it normalized it…someone shared that they tripped up the stairs at the SkyTrain station…and they felt embarrassed, you know it was like, ‘oh man’, and this is somebody that I viewed as all put together and you know? Um, so something small and silly but it was just like…you know… you see somebody trip up the stairs and you don’t think, oh my god what an idiot or like, those thoughts that you have when it happened with you.” (P5)

Another participant made an analogy between care providers demonstrating self-compassion and walking down a runway, noting that this provided a valued opportunity to observe what self-validation and self-compassion looked like:

“They (care providers) use an example of self-compassion for that situation, so kind of similar to planting a seed – showing what it could look like… and with the modelling you can, uh, you’re not required to try on the clothing, you just want to watch everyone on the runway. So you can see potential options, you don’t need to put it on yet. By seeing them model it, you can feel it, see the seams and all that, but you’re not making a purchase yet.” (P15)

Thus, participants described care providers modeling self-compassion to be an important source of validation by normalizing difficult experiences, engendering a sense of common ground, and providing insight regarding expectations for practicing self-compassion.

## Discussion

This research identified how individuals with EDs define validation and how their experiences of validation supported their recovery. Five themes, summarizing perceived validating actions performed by care providers, as well as four associated key patient experiences were identified. The use of a visual timeline allowed for relations between themes and key experiences to be depicted and suggested that some validating experiences built upon one another, while others were relevant across the recovery journey. Together, findings suggest that validating experiences increased participants’ feelings of self-acceptance and created a context of care that helped them to persevere when encountering challenges at different stages of treatment. Results have applications to multidisciplinary care providers as well as loved ones, and give rise to a number of recommendations that may be useful for those supporting individuals with EDs.

The pyramid shape of Fig. 1 offers a conceptualization of how validating experiences may build upon one another. Participants who were most ambivalent about, and least receptive to practicing self-compassion described greatest benefit from experiences that enabled them to open up, both in sharing their experiences without fear of judgment, and in cultivating a more flexible, positive view of themselves. In this high stage of ambivalence, a sense of *trust* in the relationship appeared to be most important. This may be a powerful antidote to the attachment issues that individuals with EDs often experience [10]. For patients who have very low readiness for change, care providers can use words and body language that convey interest in hearing patients’ experiences while offering a non-judgmental, compassionate appraisal of patient stories. In order to instill trust, it may be helpful for care providers to have their own mindfulness or self-compassion practice, as self-compassion in care providers is linked to empathy and emotional capacity [2, 23]. As such, it may also be helpful to work in an environment that supports self-care and well-being.

Validation that made participants feel *cared for* was engendered by interventions and a style of delivery that matched and was responsive to patient readiness for change. This matching was important at both ends of the spectrum; on the one hand patients benefitted when providers recognized their illness severity by advocating for them to receive care. On the other end, they also felt cared for when care providers supported them in recognizing that the demands of a particular treatment program exceeded their current capacity. For instance, many participants highlighted the importance of care providers conveying that it was okay to step back or change course in treatment and not being pressured or shamed for disengaging from a particular form of treatment. In order to offer validation that matches patient needs, care providers need to be aware of patient readiness by conducting ongoing readiness assessments (e.g., [24]), recognize the importance of using a collaborative stance [17, 25], and work in a clinical environment informed by practice guidelines that includes a menu of treatment options that matches intervention type to readiness (e.g., [26]). Training in Motivational Interviewing would also support care providers in providing this level of validation [27].

For participants who were actively engaged in change efforts, validation that was perceived as *empowering* was most helpful. This type of validation was characterized by interactions with care providers where participants felt seen, where the difficulty of their change efforts were acknowledged, and where setbacks were normalized and viewed as part of the recovery journey. Participants noted that in the face of struggle or relapse, care provider actions that recognized the courage and effort required for change helped them to overcome shame that might otherwise have kept them silent. That is, in being helped to talk about and learn from their experiences, they felt able to try again. In residential or inpatient programs, a clinical environment in which care providers and peers talk about and model relapses as normal and use them as an opportunity for growth might be supportive. Indeed, preparation for and anticipation of setbacks has been associated with behaviour change in the short- and long-term [28].

It should be noted that for the three aforementioned types of validation, although validating experiences built upon one another, the type that was described as most supportive at a given time could change in accordance with patient circumstances and readiness. For instance, a patient who was actively working on change and benefitting from *empowering* validation, after encountering a stumbling block and needing a change in treatment approach, subsequently most benefited from *cared for* validation that was supportive in ensuring treatment was matched to their needs. This is illustrated by the reciprocal arrows in the figure. Findings thus suggest that care providers require flexibility, the ability to conduct ongoing assessments of patient needs, and the capacity to shift the focus of validation offered throughout treatment.

Finally, across the recovery journey, participants felt validated when care providers modeled vulnerability and self-compassion. This led to participants feeling *inspired* to consider self-compassion in their own lives. Seeing self-compassion “in action” allowed for learning by example, as opposed to adhering to recommendations or teaching. An added benefit that participants stated from witnessing care providers *walk the runway* was feeling less “different” from someone they respected, which gave rise to feelings of common humanity. In considering how to incorporate *walking the runway* in practice, while care providers may vary in their level of comfort with self-disclosure, findings from this study suggest that sharing even minor examples of vulnerability and self-compassion could be of benefit to patients. Similar to recommendations for working with clinical trainees, this research therefore suggests that care providers reflect upon how to effectively model self-compassion for patients [29].

This study has some limitations. Data were collected as part of a larger project on understanding barriers and facilitators of self-compassion in EDs [21]. As such, the interview questions were not developed with the explicit intent of assessing validation, which may have impacted the number or type of themes uncovered. Nevertheless, it is noteworthy that without direct solicitation in the interview protocol, the data illustrate the salience of validation. It should also be noted that study participants had a lengthy illness duration and received treatment in a specialized program with clinicians trained to integrate self-compassion into various aspects of treatment. Finally, it is not known whether study findings would apply to individuals with a less lengthy illness history receiving care in less intensive treatment programs, or whether findings would differ in a program that places less emphasis on self-compassionate approaches to care. These are important areas for future research.

## Conclusions

This research provides the first patient-based, in depth exploration of validation, a construct described by both clinicians and individuals with EDs to play a key role in recovery. In specifically describing how validation engenders trust, as well as feeling cared for, empowered, and inspired, findings from this research provide guidelines that may assist care providers of all types (clinicians and loved ones) in helping individuals with EDs collaboratively navigate treatment challenges.

## Data Availability

To protect the confidentiality of participants as details provided in the interviews could be personally identifying, the data of this study are not publicly available.

## Abbreviations

ED: Eating disorder
P: Participant
EDE-Q: Eating Disorder Examination Questionnaire
